# The Power of Psychobiotics in Depression: A Modern Approach through the Microbiota–Gut–Brain Axis: A Literature Review

**DOI:** 10.3390/nu16071054

**Published:** 2024-04-04

**Authors:** Angela Dziedzic, Karina Maciak, Katarzyna Bliźniewska-Kowalska, Małgorzata Gałecka, Weronika Kobierecka, Joanna Saluk

**Affiliations:** 1 University of Lodz, Faculty of Biology and Environmental Protection, Department of General Biochemistry, Pomorska 141/143, 90-236 Lodz, Poland; karina.maciak@biol.uni.lodz.pl (K.M.); weronika.kobierecka@edu.uni.lodz.pl (W.K.); joanna.saluk@biol.uni.lodz.pl (J.S.); 2 Department of Adult Psychiatry, Medical University of Lodz, Aleksandrowska 159, 91-229 Lodz, Poland; 3 Department of Psychotherapy, Medical University of Lodz, Aleksandrowska 159, 91-229 Lodz, Poland; malgorzata.galecka@umed.lodz.pl

**Keywords:** microbiota–gut–brain axis, gut–brain axis, psychobiotics, probiotics, depression

## Abstract

The microbiota–gut–brain (MGB) axis is a complex communication network linking the gut, microbiota, and brain, influencing various aspects of health and disease. Dysbiosis, a disturbance in the gut microbiome equilibrium, can significantly impact the MGB axis, leading to alterations in microbial composition and function. Emerging evidence highlights the connection between microbiota alterations and neurological and psychiatric disorders, including depression. This review explores the potential of psychobiotics in managing depressive disorders, emphasizing their role in restoring microbial balance and influencing the MGB axis. Psychobiotics exhibit positive effects on the intestinal barrier, immune response, cortisol levels, and the hypothalamic–pituitary–adrenal (HPA) axis. Studies suggest that probiotics may serve as an adjunct therapy for depression, especially in treatment-resistant cases. This review discusses key findings from studies on psychobiotics interventions, emphasizing their impact on the gut–brain axis and mental health. The increasing acceptance of the expanded concept of the MGB axis underscores the importance of microorganisms in mental well-being. As our understanding of the microbiome’s role in health and disease grows, probiotics emerge as promising agents for addressing mental health issues, providing new avenues for therapeutic interventions in depressive disorders.

## 1. Introduction

Depression is a common illness worldwide, with an estimated 3.8% of the population affected globally. It is a serious medical and social problem, causing individual suffering, loss of productivity, increased health care costs, and high suicidal risk [1,2]. The number of patients receiving antidepressant treatment is increasing every year [1]. However, only 60–70% of patients suffering from this disorder respond to the standard antidepressant treatment, which means that treatment-resistant depression (TRD) can be as prevalent as 1/3 of patients with clinical depression [3,4]. Hence, it is essential to continue searching for possible pathological mechanisms in order to find new, effective, and safe therapies. Recently, nutritional interventions, including the use of probiotics to manage depression, have also become a subject of interest for researchers looking for new means of treating depression [5,6,7]. According to the latest literature reports, probiotics, with their capacity to restore microbial balance, emerge as potential agents in the treatment and prevention of mental disorders [8,9,10]. This review aims to explore the potential applications of probiotics in alleviating the symptoms of depressive disorders.

## 2. The Microbiota–Gut–Brain (MGB) Axis and Depression: Understanding the Essential Connections

**Figure 1:**
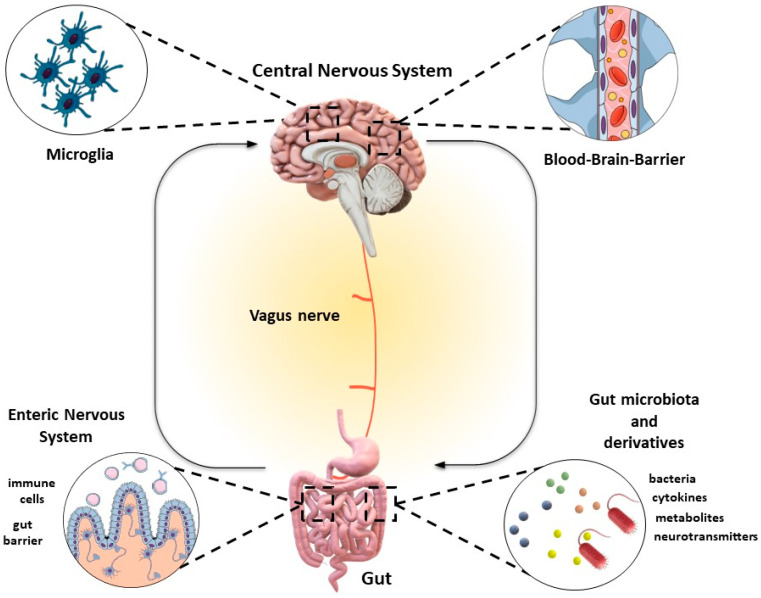
Bidirectional communication between gut microbiota and gut-brain axis (GBA). Microbiota communicates with the GBA through different mechanisms viz. direct interaction with enterocytes (enteric message), via immune cells (immune message), and via contact to neural (vagus nerve) endings (neuronal message) to influence the central nervous system (CNS). During dysbiosis a synthesis of several microbial products viz. metabolites, neurotransmitters, and cytokines gain access to the brain via the bloodstream.

Currently, depression is understood to be fueled by a complex interplay of various molecular mechanisms, primarily including a neurotransmitter reduction, abnormally stressed hypothalamus–pituitary–adrenal (HPA) axis, a decline in brain-derived neurotrophic factor (BDNF) levels, increased pro-inflammatory intestinal responses, and interaction between the intestinal microbiota and the brain via the vagus nerve [11,12,13,14,15,16]. Significantly, these conceptual frameworks are intricately linked to the interplay between the gut–brain axis (GBA) and the gut microbiota (Figure 1), commonly referred to as the microbiota–gut–brain (MGB) axis [17,18,19]. The pathway regulated by MGB affects neurobehavioral outcomes by impacting neuronal, endocrine, and immune processes [20].

According to the literature, alterations in the MGB axis have been cross-linked to a triad consisting of gastrointestinal issues, mental health problems, and neurological disorders [21]. Disturbance in the gut microbiota equilibrium, known as dysbiosis, may lead to alterations in the composition and function of the gut microbiome, thereby affecting the production of metabolites that influence neuronal activity, immunity, and intestinal inflammation [22]. 

Among patients with depression, there is an observed increase in the presence of the genus *Bacteroides* and a diminished abundance of the genera *Blautia*, *Faecalibacterium*, and *Coprococcus* [23]. Numerous in vivo studies provide solid evidence that dysbiosis may contribute to the development of depression. For example, transplantation of fecal microbiota from depressed patients into healthy rodents induced depressive behavior in them, suggesting that microbial dysbiosis precedes the onset of depression and may contribute to its development [24,25,26,27,28,29].

Consequently, while microbiome alterations may manifest early in mental disorders and potentially contribute to their onset, ongoing pathological changes may further disrupt the gut microbiota environment.

### 2.1. Gut Microbiota Metabolites in Antidepressant Mechanisms

A growing body of preclinical and clinical studies have highlighted that specific compositional changes in microbial metabolites are associated with the onset and progression of depression via regulation of the GBA [30,31,32,33,34,35,36,37,38,39,40,41,42]. The gut microbiota is an abundant source of differentiated metabolites that serve as a chemical toolbox in the communication between the intestines and CNS via GBA pathways, and their levels may partially reflect the metabolic capacity of intestine microbes [43]. These include tryptophan, γ-aminobutyric acid (GABA), histamine, serotonin, short-chain fatty acids (SCFAs), 5-hydroxytryptamine (5-HT, serotonin), dopamine, and acetylcholine (ACh) [44,45]. The multifaceted action of microbial metabolites exerts an influence on diverse mechanisms essential for maintaining mental state, including maturation of the immune [46] and neuroendocrine systems [47], regulation of nutrient metabolism, and facilitation of xenobiotic transformation [48]. Moreover, microbial metabolites play a pivotal role in safeguarding the integrity of the gut barrier, enhancing the resilience of the mucosal layer, and impeding the infiltration of harmful pathogens and toxins into the bloodstream [49].

Determining the extent to which microbial metabolism directly influences CNS activity is challenging, and distinguishing direct effects of microbial metabolites on CNS function from other communication pathways is complex. While the exploration of this fascinating relationship is still ongoing, a set of fundamental mechanisms have been elucidated, offering insights into the profound impact of microbiota on CNS homeostasis and neurological disorders [20].

#### 2.1.1. Short-Chain Fatty Acids (SCFAs)

Short-chain fatty acids (SCFAs), including butyrate, propionate, and acetate, are a subtype of fatty acids notably abundant in the proximal colon due to the fermentation of partial and non-digestible polysaccharides like dietary fiber or resistant starch. They significantly influence emotional states and cognition through G-protein-coupled receptors, suggesting a connection between SCFAs and the underlying mechanisms of depression. SCFAs may cross the BBB and reach the CNS, allowing for intestinal flora to interact with a host’s brain [50].

According to recent research, the most implicated bacterial strains in SCFAs production are Bacteroidetes and specific species of the Firmicutes phylum [51,52]. *Eubacterium rectale*, *Roseburia faecis*, *Eubacterium hallii*, and *Faecalibacterium prausnitzii* are key butyrate producers in the gut, while *Veilonella*, *Lactobacillus*, *Bacteroides*, and *Propionibacterium* are notable for propionate production. Acetate production, however, is typically carried out by various bacterial classes [53].

SCFAs serve as the primary energy source for colonocytes, contribute to the maintenance of the intestinal barrier by promoting mucin production and strengthening tight junctions, regulate the inflammatory response, affect intestinal motility [52], and control hormones involved in appetite regulation, peptide YY (PYY), and glucagon-like peptide 1 (GLP-1) [54]. Another mechanism by which SCFAs regulate systemic functions is through the inhibition of histone deacetylase (HDAC) activity, thus promoting the acetylation of lysine residues present in nucleosomal histones throughout various cell populations. SCFAs may also be absorbed into the bloodstream and serve as a substrate for metabolites, such as serotonin and GABA, and can also be involved in gluconeogenesis and lipogenesis [55]. Recent investigations underscore the fact that SCFAs emerge as pivotal actors implicated in stress-related pathologies, notably encompassing anxiety- and depression-related phenotypes [56]. Furthermore, increased SCFAs ameliorate neuroinflammation and stimulate the production of BDNF involved in brain neuroplasticity [57].

The amounts of SCFAs in the feces of depressive mice and humans have been found to be decreased in most cases [58,59,60]. Fecal microbiota transplantation (FMT) is believed to be an effective method for reconstructing intestinal microbiota and host function. Multiple in vivo studies have shown that FMT of stressed mice onto germ-free mice leads to depression-like behavior in the recipient mice [61,62]. Furthermore, the effectiveness of SCFA supplementation in ameliorating the adverse effects of chronic psychosocial stress on the exhibition of anxiety- and depressive-like phenotypes, along with concomitant alterations in behavior, was demonstrated [63,64,65].

Recent studies also provide some details on the epigenetic modifications affected by SCFAs, especially butyrate, which results in altered gene expression in the gut and CNS. For example, sodium butyrate has been reported to ameliorate intestinal epithelium barrier injury, attenuate deficits in social behavior, and improve the neuroprotective function of microglia [66,67,68,69].

The homeostasis of microglia in the CNS has also been found to be regulated by SCFAs. Mice lacking the SCFAs receptor FFAR2 exhibited microglial defects similar to those observed under GF conditions. These results suggest that the host’s resident bacteria play a crucial role in controlling microglial maturation and function [70].

#### 2.1.2. Kynurenine Pathway Metabolites and Neurotransmitters

Another group of metabolites produced by the microbiota are tryptophan derivatives synthesized via the kynurenine pathway [71]. Five bacterial phyla, namely Firmicutes, Bacteroidetes, Actinobacteria, Proteobacteria, and Fusobacteria, have been implicated in tryptophan metabolism. For instance, formulations with potential psychological benefits often incorporate two psychobiotic strains: *Lactobacillus helveticus* R0052 and *Bifidobacterium longum* R0175. Studies have shown that supplementation with *Lactobacillus helveticus* R0052 and *Bifidobacterium longum* R0175 can alleviate symptoms of anxiety and depression in healthy volunteers. Bacteria involved in tryptophan metabolism can be, e.g., *Enterobacter aerogenes*, *Clostridium limosum*, *Clostridium tetani*, *Clostridium lentoputrescens*, *Clostridium bifermentans*, and *Clostridum melanomenatum*, belonging to the Firmicutes phyla, as well as members of Bacteroidetes, Fusobacteria, and Proteobacteria [72]. Certain gut bacteria, such as *Lactobacillus*, *Lactococcus*, *Streptococcus*, and *Klebsiella*, increase the gene expression of tryptophan synthetase, leading to the augmented production of serotonin [71]. Furthermore, studies have reported that *Akkermansia muciniphila* and its extracellular vesicles affected the mRNA expression of genes involved in serotonin signaling/metabolism in the hippocampus and the colon through the GBA [73,74,75,76].

There are two pivotal processes that the MGB axis involves: (i) the conversion of tryptophan into the neurotransmitter serotonin, known for its beneficial effects on brain and gut functions, and (ii) the metabolic pathways of tryptophan leading to kynurenine, tryptamine, and indole, which exert neuroendocrine and immune-modulatory effects [71]. Under the influence of the indoleamine 2,3 dioxygenase (IDO) enzyme, tryptophan is transformed into kynurenine, quinolinic acid, and 3-hydroxy-kynurenine, which may lead to tryptophan and 5-HT depletion, intensifying the symptoms of affective diseases and depression [77]. Taking these relationships into account, one hypothesis regarding the development of affective disorders involves inflammation within the human body [78].

Monoamines derived from tryptophan, such as tryptamine and serotonin, promote gut motility by activating the serotonin receptors 5-HT4R and 5-HT3R [79]. Within the intestinal tract, enterochromaffin cells synthesize serotonin with the help of the enzyme tryptophan hydroxylase 1 (TPH1), and *Tph1* mRNA expression is promoted by the gut microbiota, specifically by SCFAs [56]. Then, serotonin is taken up by the intestinal epithelium through the serotonin-selective reuptake transporter (SERT), thereby influencing the development and signaling of the enteric nervous system (ENS). Recent findings demonstrate that SCFAs derived from the microbiota play a role in regulating the intestinal serotonergic system, influencing the activity and expression of SERT, as well as of the 5-HT1A, 5-HT2B, and 5-HT7 receptors [80].

Most of the serotonin is generated in the gut, and the levels of 5-HT in the gut do not have a direct impact on the levels of 5-HT in the brain due to the inability of 5-HT to pass through the BBB. However, Clarke et al. have demonstrated that gut microbiota actually has an impact on the brain serotonergic system, as male GF mice exhibited a notable increase in the levels of 5-HT and its primary metabolite, 5-hydroxyindoleacetic acid, in the hippocampus when compared to control animals with a conventional microbiota [81]. These mechanisms collectively represent a potential antidepressant pathway mediated by the gut microbiota and its interaction with the serotonergic system.

Apart from serotonin, gut bacteria can influence the production and regulation of neurotransmitters, such as dopamine, acetylcholine, and GABA, impacting their availability in the brain [82]. Specific bacterial species, such as *Enterococcus faecalis* and *Enterococcus faecium*, are involved in the production of dopamine by decarboxylation of levodopa (L-dopa) [83,84]. Bacteroides can produce GABA directly through the glutamate decarboxylase (GAD) system as a protective reaction to acid stress [85]. In addition, *Bifidobacterium* and *Lactobacillus* have also been reported to be capable of producing GABA [86], enhancing the inhibitory pathway in brain networks. GABA produced in the gut can influence GABAergic signaling in the enteric nervous system, affecting gut motility [85]. Certain gut bacteria, such as *Lactobacillus plantarum* [87] and *Bacillus subtilis* [88], can produce acetylcholine precursors and affect acetylcholine metabolism.

### 2.2. Gut Microbiota and CNS Functioning and BBB Permeability

The gut microbiota can influence the integrity and function of the BBB, potentially impacting the access of various substances, including neuroactive factors, to the brain.

Furthermore, researchers have recently uncovered the connection between stress, gut microbiota, and disruptions in the BBB. It is revealed that induced chronic social defeat stress (CSDS) and learned helplessness (LH) in rodents lead to compromised BBB integrity by reducing the expression of tight junction proteins, such as claudin-5, leading to a massive infiltration of pro-inflammatory cytokines, such as IL-6, into the CNS [89]. Braniste et al. have reported that BBB permeability and down-regulated tight junction proteins caused by GF conditions may be decreased through postnatal colonization with butyrate-producing *Clostridium tyrobutyricum* or with acetate- and propionate-producing *Bacteroides thetaiotaomicron* [50]. Also, the relationship between the integrity of the blood–cerebrospinal fluid barrier and the gut microbiota has been reported, as GF mice exhibit decreased tight junction protein in the area of choroid plexus epithelium [90].

The main neuronal communication pathway involving the autonomic nervous system runs via the vagus nerve, a vital conduit linking the intestines and the ENS with the brainstem. This pathway facilitates the transmission of signals associated with gastrointestinal function to the CNS. The sensory fibers of the vagus nerve, equipped with specialized receptors in the intestinal lining, can detect signals triggered or produced by the microbiota. This pathway can detect the presence of specific metabolites, microbial by-products, and neurotransmitters from the ENS, cytokines, or gut hormones [91]. For example, the vagus nerve possesses transmembrane molecular sensors, including GPCRs [92], such as GPR41 and GPR43, activated by SCFAs. The production of neurotransmitters, such as serotonin, GABA, and glutamate, is influenced by the binding of microbial metabolites to specific receptors in vagal sensory neurons. Neurotransmitters produced in response to microbial signals can influence neural signaling within the ENS and the gut. When the gut microbiota generates metabolites or produces signaling molecules, the sensory fibers of the vagus nerve can detect these signals and transmit them into the brain, conveying information about the gut’s status and microbial activity [91].

### 2.3. Dysregulated MGB Axis in Depression: Chronic Stress Response

Stress significantly disturbs intestinal homeostasis and shifts the gut microbial composition [93]. In humans, it has been shown that chronic stress elevates *Faecalibaculum* [94] and *Clostridium* [95], alongside producing a reduction in *Lactobacillus* and *Bifidobacterium* [96]. Thus, a comprehension of the exact taxonomic shifts within the microbial composition triggered by stress holds significant relevance in guiding the selection of suitable prebiotics and probiotics. Recent research on animal models of chronic stress has revealed a link between the composition of the MGB axis and susceptibility and resilience to stress [97,98].

In a mouse model of permanent stress, higher levels of *Bifidobacterium* were found in stress-resistant mice, while in susceptible mice *Bifidobacterium* levels were below the detection limit. Moreover, oral consumption of *Bifidobacterium* significantly increased the number of resistant mice. A far-reaching conclusion allows us to assume that *Bifidobacterium* supplementation could prevent the occurrence of depression caused by stress in humans [99].

The latest study conducted by He et al. (2024) has revealed a correlation between stress resilience in mice and the composition of gut microbiota. Stress-resistant mice exhibited higher levels of *Lactobacillus* and *Akkermansia*, but lower levels of *Bacteroides*, *Alloprevotella*, *Helicobacter*, *Lachnoclostridium*, *Blautia*, *Roseburia*, *Colidextibacter*, and *Lachnospiraceae* [100]. In this study, mice sensitive to stress displayed increased gut permeability, heightened immune responses within the colon, and elevated serum levels of pro-inflammatory cytokines. Furthermore, their hippocampal region exhibited heightened microglial activation, disrupted microglia–neuron interactions, and reduced synaptic plasticity. Fecal microbiota transplantation from stress-sensitive mice to naïve counterparts disrupted microglia–neuron interactions and impaired synaptic plasticity in the hippocampus, resulting in depression-like behavior post-stress exposure [100]. These findings propose that the gut microbiota may modulate resilience to chronic psychological stress through the regulation of microglia–neuron interactions within the hippocampus.

Recent studies have suggested that the MGB axis contributes to the development of depression through its interaction with the vagus nerve, and that vagotomy influences the effect of fecal microbiota transplantation on stress-related behavior [101]. Acute stress triggers the activation of the HPA and sympathetic adrenal–medullary (SAM) pathways, resulting in the excessive production of stress hormones, predominantly cortisol and catecholamines. Increased levels of stress hormones activate SAM and stimulate the processes of lipolysis and glycogenolysis, leading to vasoconstriction and enhanced blood pressure, while chronic activation of the HPA axis leads to alterations in the expression levels of the inflammatory cytokines. Furthermore, stress-related hormones may contribute to direct gut and oral microbiome alterations [102].

Interestingly, administering specific strains of *Lactobacillus* or *Bifidobacterium* has been found to lower cortisol levels in individuals experiencing acute or chronic stress. This was confirmed, for example, in studies on *Lactobacillus intestinalis* and *Lactobacillus reuteri* [103]. Meanwhile, treatment with *Lactobacillus rhamnosus* resulted in reduced anxiety-like behavior, alleviated dendritic cell activation, and increased IL-10+ Tregs levels [104]. A preclinical study demonstrated that oral supplementation of *Bifidobacterium* can induce stress resilience. Yang et al. have demonstrated that *Bifidobacterium* at a dose of 10 mg/kg/day for 20 days imparts resilience against CSDS in mice and mitigates the symptoms of depression [99]. On the other hand, it has been suggested that *Faecalibaculum rodentium* and the MGB axis, mediated by the subdiaphragmatic vagus nerve, also play crucial roles in stress susceptibility and resilience. *Faecalibaculum rodentium* caused depression-like behaviors and inflammation in Ephx2 KO mice, which was prevented by subdiaphragmatic vagotomy [105]. Furthermore, *Clostridium perfringens* has been found to reduce the viability of neurons and also inhibit the release of neurotransmitters by reaching the brain through the disrupted BBB [106].

### 2.4. Gut Microbiota and Inflammation

The gut microbiota exerts a profound influence on the immune system, thus shaping inflammatory responses and states within the brain, orchestrating complex interactions that modulate immune responses, promoting tolerance to commensal microorganisms, and contributing to the maintenance of immune homeostasis. A resilient and diverse gut microbiome is indispensable for robust immune function and appropriate immunoregulatory mechanisms [107].

Psychiatric conditions have also shown associations with variations in gut microbiota, and are linked to either anti-inflammatory or pro-inflammatory functions. A meta-analysis involving 1519 patients diagnosed with various psychiatric disorders (such as major depressive disorder (MDD), bipolar disorder, psychosis, schizophrenia, anorexia nervosa, anxiety disorders, obsessive compulsive disorder (OCD), post-traumatic stress disorder (PTSD), and attention-deficit/hyperactivity disorder (ADHD)) revealed consistent differences in microbial diversity. Specifically, individuals with depression displayed reduced levels of beneficial, anti-inflammatory bacteria like *Faecalibacterium* and *Coprococcus*, while experiencing increased levels of pro-inflammatory bacteria such as *Eggerthella*, compared to healthy controls. This suggests that depression is characterized by a depletion of bacteria that produce anti-inflammatory compounds like butyrate, while simultaneously exhibiting an enrichment of pro-inflammatory microbial genera [108,109].

The main cause of the systemic inflammatory response is enhanced intestinal permeability, a condition commonly known as a leaky gut syndrome (LGS) [110]. The mechanisms engaged for LGS development are primarily gut microflora disturbances, breakdown of the intestinal barrier, and damage of enterocytes, leading to systemic inflammation, which is critical for depression pathophysiology [111]. As a result of LGS development, the translocation of Gram-negative bacteria endotoxins (LPS, lipopolysaccharide) into the bloodstream triggers the immune response [112]. LPS is a potent stimulus for pro-inflammatory molecule synthesis by a variety of types of effector cell, including immune cells (mainly macrophage, mast cells, and T-cells) and the CNS cells (such as astrocytes, microglia, and neurons) [113]. Furthermore, due to the increase in BBB permeability, LPS represents a molecular link between gut dysbiosis and neuroinflammation [114]. Pro-inflammatory molecules generated by LPS-induced cells include eotaxin, histamine, heparin, chymase, carboxypeptidase, tryptase, chondroitin sulfate, interleukins (ILs) (IL-1β, IL-3, IL-4, IL-6, IL-8, IL-9, IL-10, IL-13, IL-16, IL-18, and IL-25), tumor necrosis factor α (TNF-α), macrophage chemotactic peptide (MCP)-1, granulocyte–macrophage colony-stimulating factor (GM-CSF), stem cell factor (SCF), platelet activating factor (PAF), leukotriene 4 (LTC4), as well as RANTES (regulated on activation of normal T cell-expressed and secreted protein) [115].

All of the pro-inflammatory molecules, due to their small size, can freely pass through the BBB via various mechanisms such as passive diffusion through the leaky regions of the gut barrier, active transport, or through the nerve fibers (vagus or trigeminal nerves) [116]. What is more, increased concentrations of pro-inflammatory mediators may affect the activity of essential enzymes of the kynurenine pathway [117], such as IDO [15].

It is known that gut microbiota variants with a pro-inflammatory phenotype promote an immune cell over-activation as a response to signals recognized not only by the Toll-like receptors (TLRs) due to the action of bacterial LPS, but also to the action of other microbial derivatives. Intestinal epithelial cells express a variety of pattern recognition receptors (PRRs) that bind to specific microbial-derived motifs, such as pattern-associated molecular patterns (PAMPs) and damage-associated molecular patterns (DAMPs) exhibited by pathogens invading the host organism [118]. In the intestine, PRRs play a crucial role in regulating the composition of the resident microbiota. PRRs induce inflammatory signaling, promote cell proliferation in response to mucosal damage, and contribute to the elimination of pathogens and damaged or dead cells. PRRs also mediate the antigen-specific adaptive immune response and increase the amount of IgA produced by triggering immunoglobulin isotype switching in B-cells [119]. When PRR receptors are stimulated, the communication via the peripheral and central inflammatory pathways is mediated by many inflammation-associated proteins, including inflammasomes, cytosol multiprotein complexes which are induced in response to PRR engagement. Their assembly is initiated by the activation of the intracellular NOD-like receptors NLRP1, NLRP3, NLRC4, or AIM2, which all are geared towards responding to microbial products emerging in the cytoplasm [120]. In the gut, dysfunctional inflammasome formation is linked, among others, to immunopathology in the GBA and chronic gut inflammation [121,122].

Examples of bacteria that demonstrate an impact on both intestinal and neurological health are presented in Table 1.

## 3. Impact of Psychobiotics and Healthy Dietary Patterns on Depressive Disorders

**Figure 2:**
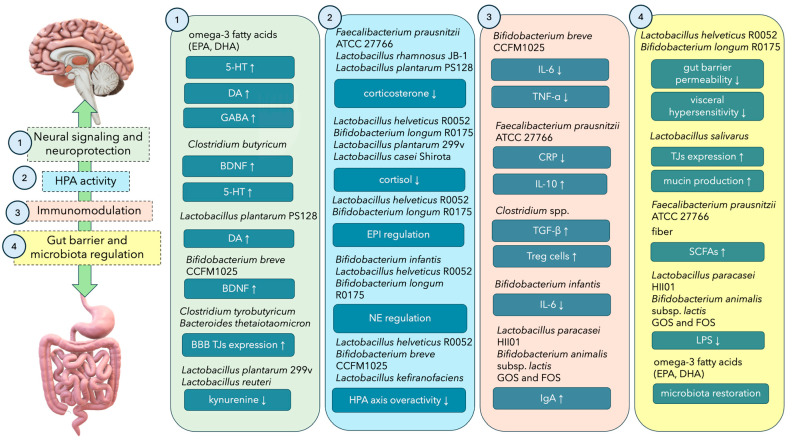
Overview of the pathways of antidepressant effects on the GBA axis exerted by probiotics and prebiotics. 5-HT—5-hydroxytryptamine; DA—dopamine; GABA—γ-aminobutyric acid; BDNF—brain-derived neurotrophic factor; BBB—blood–brain barrier; EPI—epinephrine; NE—norepinephrine; HPA axis—hypothalamic-pituitary-adrenal axis; IL—interleukin; TNF-α—tumour necrosis factor α; CRP—C-reactive protein; TGF-β—transforming growth factor β; IgA—immunoglobulin A; TJs—tight junctions; SCFAs—short-chain fatty acids; LPS—lipopolysaccharide.

Probiotic bacteria that benefit mental health when consumed in adequate amounts are called psychobiotics. This name was first used by T.G. Dinan in 2013 [147]. Psychobiotics positively affect the parameters of the intestinal barrier and modulate the immune response in the gut-associated lymphoid tissue (GALT) area, which is involved in the development of inflammation. The beneficial effects of psychobiotics include lowering cortisol levels and HPA axis activity, as well as modulating vagus nerve stimulation [148]. Figure 2 presents an overview of the most common pathways through which microbes and prebiotics may exert antidepressant effects on the GBA.

The influence of the gut microbiota on inflammatory processes or the stress axis, described above, point to its importance in the pathogenesis of depression, in which these very elements play a key role.

### 3.1. Preclinical Studies on Animal Models

In 2006, the first probiotic preparation containing *Lactobacillus helveticus* R0052 and *Lactobacillus rhamnosus* R0011 strains was tested in a chronic psychological stress model. In subsequent years, through in vivo studies using a strategy of infecting mice and rats with intestinal pathogens and then studying their behavior, scientists had established that the composition of the intestinal microbiota influences the behavior of the animals studied [149,150,151,152]. Thus, any permutation in the composition of the gut microbiome results in the production of LPS by microorganisms, which in turn activates inflammatory responses. The pro-inflammatory cytokines produced send signals to the vagus nerve, thus connecting to the stress axis, which consequently affects the behavior [153,154,155].

Behavioral tests have shown that probiotic therapy reduces the intensity of depressive behavior in rodents. Researchers have concluded that the introduction of probiotics was effective in reducing behavioral deficits and emotional memory processing in a model of depression caused by myocardial infarction [156]. In 2016, Callaghan et al.—using the MS (maternal separation) test—found that probiotic administration restored normal development and emotional stability in rats experiencing early developmental stress [157,158]. Additionally, supplementation with *Lactobacillus helveticus* R0052 and *Lactobacillus rhamnosus* R0011was effective in reducing the generational effects of stress in infant rats. Ait-Belgnaoui et al. (2014) showed that *Lactobacillus helveticus* R0052 supplementation reduces deficits related to plasticity and neurogenesis, which are caused by chronic stress [139], and in consequence decreases the activity of the HPA axis and autonomic system due to reduced cortisol and catecholamine levels. Probiotics have been proven to improve the tightness of the intestinal barrier; however, this benefit was not observed with another probiotic species, *Lactobacillus salivarus*. A more recent study by Ait-Belgnaoui et al. (2018) found that the administration of probiotics formulated with *Lactobacillus helveticus* R0052 and *Bifidobacterium longum* R0175 significantly reduced visceral hypersensitivity caused by chronic stress [159], which correlated with a decrease in corticosterone, norepinephrine, and epinephrine levels.

Studies have also identified the positive effects of psychobiotics using distinct strains of probiotic bacteria. In a study by Liu et al. (2016), naïve adult mice and mice under stress in early life were administered 1 × 10^9^ CFU of *Lactobacillus plantarum* PS128 [160]. An open field test showed that the administration of *Lactobacillus plantarum* PS128 increased psychomotor activity in both stressed and naïve adult mice in early life. In a cross maze test, *Lactobacillus plantarum* PS128 helped reduce anxiety behaviors in naïve adult mice. These activities were not observed in stressed mice in early life. The study showed a noticeable effect on immune system parameters. The supply of psychobiotics used in the study reduced serum corticosterone levels in both the primary and stressed states in the mice that were stressed early in life. However, no effect was observed in naïve mice. The levels of inflammatory cytokines also decreased, while serum anti-inflammatory cytokines increased in the mice stressed early in life. The dopamine levels in the prefrontal cortex were elevated in both treated groups, while the serotonin levels were elevated only in the naïve adult mice [160]. Another study confirming the beneficial effects of psychobiotics on animal models of depression conducted by Tian et al. [161] has identified the psychobiotic potential of *Bifidobacterium breve* CCFM1025. In the experiment, C57BL/6J mice were administered 0.1 mL/10 g body weight of *Bifidobacterium breve* CCFM1025 suspension at a concentration of 1 × 10^9^ CFU/mL daily for 5 weeks. The study showed that treatment with *Bifidobacterium breve* CCFM1025 helped reduce anxiety and depressive behaviors. Inflammation caused by the HPA axis reactivity was alleviated, resulting in reduced glucose receptor expression and hypocamic receptor expression. Reduced levels of IL-6 and circulating TNF-α were also determined. In addition, an upregulation of BDNF was noted. Supplementation with the aforementioned psychobiotic restored the balance of the intestinal microflora, particularly in terms of the ratio of Actinobacteria to Proteobacteria.

In a similar preclinical study, Hao et al. investigated the potential of *Faecalibacterium prausnitzii* ATCC 27766 to reduce anxiety and depression symptoms [123]. A total of 60 male rats were divided into three groups: untreated rats, stressed rats, and stressed rats + *Faecalibacterium prausnitzii* group. The rats were fed 200 μJ of phosphate-buffered saline (PBS) solution containing 1 × 10^9^ CFU of *Faecalibacterium prausnitzii* ATCC 27766 daily for 4 weeks via oral probe. The group treated with psychobiotics had higher cecal SCFAs levels and higher plasma IL-10 levels. *Faecalibacterium prausnitzii* ATCC 27766 supplementation prevented stress-related effects such as the release of corticosterone, C-reactive protein (CRP), and IL-6 [123]. Another study on the same animal model used young adult rats separated from their mothers. Each day, the scientists orally administered 1 × 10^10^ live *Bifidobacterium infantis* 35,624 bacterial cells in 100 mL of drinking water, or citalopram [124]. The animals were then subjected to a forced swim test and cytokine concentrations, brain monoamine levels, and HPA axis measurements were determined. They found normalization of the immune response, reversal of behavioral deficits, and restoration of basal brain norepinephrine concentrations [124].

Bravo et al. (2011) have evaluated whether *Lactobacillus rhamnosus* JB-1 mediated direct effects on the GABAergic system and examined all behaviors related to GABAergic neurotransmission and stress responses [61]. In the study, mice were orally administered *Lactobacillus rhamnosus* JB-1 containing 1 × 10^9^ CFU for 28 consecutive days. The study confirmed that probiotic administration decreased corticosterone and reduced behaviors associated with depression and anxiety [61].

### 3.2. Clinical Human Studies on Psychobiotics

Successful preclinical results have also been translated into clinical studies. The bacterial strains that have been most studied for their anti-anxiety and antidepressant effects are *Lactobacilli*, *Streptococci*, *Bifidobacteria*, *Escherichia,* and *Enterococci*, and their growth/expansion can be supported by dietary fibers (prebiotics), such as fructooligosaccharides and galactooligosaccharides [107,130]. For this reason, the definition of psychobiotics has been expanded to include not only live microorganisms, but also their metabolites, inactivated cells (postbiotics), and other molecules that support the development of psychobiotic strains in the intestine (prebiotics), such as dietary fiber, which, by interacting with the human intestinal microflora, can also provide health benefits [106].

Studies have confirmed that the use of products or supplements rich in live bacterial cultures, such as *Bifidobacterium bifidum*, *Bifidobacterium lactis*, *Lactobacillus acidophilus*, *Lactobacillus brevis*, *Lactobacillus casei*, *Lactobacillus salivarius*, or *Lactobacillus lactis*, has a positive effect in terms of improving overall well-being and the severity of depressive symptoms after about 3–4 weeks of their use, depending on the initial severity of the symptoms related to patients’ mental functioning [131].

Takada et al. (2016) have studied the effects of the *Lactobacillus casei* Shirota strain on gut–brain interactions under stress in both human and animal models [125]. In the clinical part of the study, healthy medical students preparing for an important exam were given fermented *Lactobacillus casei* Shirota milk (1 × 10^9^ CFU/mL) or a placebo of unfermented milk for 8 weeks. During the experiments, participants were asked to complete daily and weekly questionnaires regarding any physical complaints. In addition, saliva samples were collected at the beginning of the study, 6 weeks after the intervention, the day before the study, and immediately after the study (on the same day as the study) to measure cortisol levels. The authors observed that the change in cortisol levels from baseline was significantly lower in the psychobiotic group than in the placebo group on the day before the study. As for the incidence of somatic symptoms, the *Lactobacillus casei* Shirota-supplemented group had a significantly lower incidence of flu-like symptoms and abdominal symptoms compared to the placebo group, which once again demonstrated the positive role of psychobiotics [125].

The interest in probiotics has led to further findings supporting their use in reducing anxiety disorders. In another study performed to determine the effect of daily supplementation with *Lactobacillus casei* Shirota on stress and anxiety in athletes, 20 male athletes participated in a study in which they received a probiotic containing 1 × 10^9^ CFU of the *Lactobacillus casei* Shirota strain for 8 weeks. Anxiety and perceived stress were measured at the beginning of the study, at week 4, and at week 8 using an anxiety inventory and a scale for the level of stress experienced. Daily supplementation with psychobiotics was found to significantly reduce the effects of anxiety as a cognitive state, somatic state, and perceived stress level [126]. The study did not measure biological parameters, so the mechanism of action by which the strain selected for the study eliminated anxiety behaviors was not identified [127]. The results of the study by Zhu et al. (2023) also indicate that the intervention with *Lactobacillus plantarum* JYLP-326 is an effective strategy to alleviating anxiety, depression, and insomnia in anxious college students [162].

The study by Zhang et al. (2021) concluded that the consumption of *Lacticaseibacillus paracasei* strain Shirota (LcS) daily for 9 weeks significantly improves the constipation and depressive symptoms in patients with depression, although the changes in depressive symptoms were not significant between groups. Additionally, IL-6 levels were significantly lower in the LcS group than in the placebo group [163].

Akkasheh et al. (2016) have performed a randomized, double-blind, placebo-controlled clinical trial on 40 depressed patients who were randomly allocated into two groups to receive either probiotic supplements (*n* = 20) or a placebo (*n* = 20) for 8 weeks. The probiotic capsule consisted of *Lactobacillus acidophilus* (2 × 10^9^ CFU/g), *Lactobacillus casei* (2 × 10^9^ CFU/g), and *Bifidobacterium bifidum* (2 × 10^9^ CFU/g). Those who received the probiotic supplements had significantly decreased Beck Depression Inventory (BDI) total scores (−5.7 ± 6.4 vs. −1.5 ± 4.8, *p* = 0.001) compared with the placebo-receiving group [164].

The use of dietary supplementation with the probiotic strain *Clostridium butyricum* (CBM588) in combination with antidepressants contributed to a significant reduction in scores (e.g.,  ≥50% reduction in total score) on the Hamilton rating scale for depression (HAMD-17) for the BDI in a group of patients diagnosed with treatment-resistant major depressive disorder compared to a placebo-treated control group [128].

The inclusion of probiotic therapy based on the use of a psychobiotic preparation containing *Lactobacillus helveticus* R0052 and *Bifidobacterium longum* R0175, as well as *Lactobacillus plantarum* 299v, can contribute to a significant reduction in the HPA response to stress [119,134], which was observed as reduced salivary cortisol levels [134]. Moreover, the regular consumption of probiotic yogurt, which included *Lactobacillus acidophilus* LA5 and *Bifidobacterium lactis* BB12 strains, as well as dietary supplementation with a multi-strain probiotic containing strains of *Lactobacillus casei*, *Lactobacillus acidophilus*, *Lactobacillus rhamnosus*, *Lactobacillus bulgaricus*, *Bifidobacterium breve*, *Bifidobacterium longum*, and *Streptococcus thermophilus*, contributed to significant improvements in health parameters and Depression Anxiety and Stress Scale (DASS) scores in 70 petrochemical workers after 6 weeks. Interestingly, the effects were not observed in those who consumed traditional yogurt [135].

This effect also turned out to be pronounced for lactic acid bacilli from Italian cheeses, especially *Lactobacillus paracasei* PF6, *Lactobacillus delbrueckii* subsp. *bulgaricus* PR1, *Lactococcus lactis* PU1, and *Lactobacillus brevis* PM17, as well as strains isolated from traditional fresh/raw milk products, mainly *Lactococcus lactis* and *Streptococcus thermophilus*, which can produce significant amounts of GABA during lactic fermentation [136,137]. On the other hand, a study evaluating the results of 24-week postbiotic supplementation in the form of inactivated cultures of *Lactobacillus gasseri* CP2305 showed that the treatment used, by affecting the composition of the intestinal microbiota, contributed to a reduction in feelings of anxiety and had a positive effect on sleep quality in adults subjected to prolonged stress, compared to placebo [138].

There are also studies that question the significant effect of probiotics on the occurrence and severity of symptoms related to psychological functioning, i.e., mood, feelings of perceived anxiety and stress, as well as other psychological disorders in people aged 24–50. The duration of probiotic supplementation in these studies ranged from 3 to 24 weeks, and they mainly involved taking a probiotic preparation once a day (85.2%), in powder form (35.2%), in liquid form, such as milk or yogurt (22.2%), or in capsule form (24.1%), containing one (53.7%) or multiple strains, ranging from 2.3 × 10^7^ to 1.4 × 10^11^ CFU per day. The effect of the selected probiotics was observed in combination with other substances, such as vitamin D, selective serotonin reuptake inhibitors (SSRIs), or lactalbumin. Therefore, the exact mechanism needs to be better understood in order to accurately assess the pathways of direct effects of psychobiotics on depressive disorders and their efficacy [139].

A meta-analysis by Zhang et al. (2023), which analyzed thirteen studies with 22 treatment and control groups, has confirmed that agents that manipulate the gut microbiota, such as probiotics, could become a new approach to treating patients with mild to moderate depression. The superiority of probiotics over prebiotics and symbiotics in antidepressant effects is apparent. Interestingly, biological sex was significant in the response to treatment. Studies containing a lower percentage of females (<70%) found a larger reduction in depressive symptom scores [114].

There is also a study that examined the administration of a synbiotic, which is the fusion of a probiotic with a prebiotic. In this study, the symbiotic administered to participants (32 volunteers) for 12 weeks contained a formulation of 1 × 10^10^ CFU/day of live probiotic strains, including 5.0 × 10^9^ CFU of *Lactobacillus paracasei* HII01 and 5.0 × 10^9^ CFU of *Bifidobacterium animalis* subsp. *lactis* (a multi-strain probiotic), and 10 g of a prebiotic consisting of 5 g of galactooligosaccharides (GOS) and 5 g of fructooligosaccharide (FOS). After the supplementation, it turned out that among the participants who were in the group defining themselves as non-stressed, the administration of synbiotics significantly reduced the level of tryptophan, while it increased the level of TNF-α, 5-hydroxyindoleacetic acid (5-HIAA), as well as the amount of short-chain fatty acids (SCFAs), including acetate and propionate. Importantly, the levels of cortisol (stress hormone) and LPS were reduced in both groups, stressed and non-stressed, while the levels of the anti-inflammatory mediator IL-10 and immunoglobulin A (IgA) were significantly increased, which may suggest that synbiotic supplementation may help reduce negative feelings in stressed participants by modulating the HPA, and that it is also able to reduce inflammation in the body. However, it should be noted that this effect may be less noticeable in people who do not experience stress. In the case of this study, the administration of synbiotics to non-stress-experiencing participants did not have as much benefit in terms of improved mood, but it still showed positive effects on the levels of probiotic-derived metabolites, such as SCFAs and tryptophan catabolism [140].

The effects of the various bacterial strains included in the formulation of pro-/postbiotics or foods obtained by fermentation, along with the dose and the health effect described, are summarized in Table 2.

Prebiotics are ingredients that are not digested, and they stimulate the growth or activity of beneficial bacteria present in the large intestine. In the study by Kazemi et al. (2019) in which 27 depressed patients received a prebiotic (galactooligosaccharide) for 8 weeks, no significant antidepressant effect (measured in BDI score) due to its supplementation was seen. Although the administration of prebiotics alone does not significantly reduce depressive symptoms, the addition of a prebiotic inulin to a probiotic shows a positive effect on psychological outcomes and inflammatory biomarkers [179,180,181].

Fiber has also been shown to be significant in regulating the GBA and is an important part of a balanced diet. Studies have confirmed the link between dietary fiber intake and the risk of depression. The results of an observational study of more than 69,000 postmenopausal women showed that a high intake of dietary fiber (about 21 g per day) at baseline resulted in a 14% lower risk of depressive symptoms (3 years later). Interestingly, those with a relatively lower fiber intake in that study (about 14 g per day) had a reduced risk of only 4% [182]. It is also interesting to note that an increased intake of soluble fiber, but not insoluble fiber, was associated with a lower incidence of depressive symptoms in both men and women. In addition, increased dietary fiber from vegetables, including soy-based products, was associated with a lower risk of depression in both genders [183].

Dietary fiber has been recognized as a key component of a healthy diet, and, unlike other nutrients such as carbohydrates, fats, or proteins, is not digested by the human digestive system. Consequently, it passes, intact, to further structures of the digestive tract, eventually reaching the large intestine where it is partially or completely fermented by the bacteria residing there, which are also known as the intestinal microflora. Fermentation of dietary fiber by the gut microbiota results in the production of biologically active molecules such as SCFAs, including acetate, propionate, and butyrate. Two of the most dominant bacteria in the microbiota are the Bacteroidetes, which produce greater amounts of acetate and propionate, and the Firmicutes, which produce more butyrate [184]. The bacterial production of SCFAs is considered by many researchers to be one of the main mechanisms through which dietary fiber can affect inflammation and thus the risk of diseases associated with it (including depression).

Omega-3 fatty acids are an important building block of cell membranes, especially those present in brain structures, as it turns out that the gray matter of the brain in its structure can contain up to 50% polyunsaturated fatty acids, 33% of which are omega-3 fatty acids, mainly docosahexaenoic acid (DHA) and eicosapentaenoic acid (EPA). Given the important role of DHA and EPA in brain processes, it is not surprising that their deficiencies have been linked to disorders of brain function, as well as an insufficient production of neurotransmitters (especially serotonin, norepinephrine, and dopamine). For example, DHA deficiencies have been linked to an impaired transmission of serotonin, norepinephrine, and dopamine, which in turn are important in the development of mood disorders found in depression, among others. Interestingly, supplementation with omega-3 fatty acids can help preserve and restore the proper functioning of the microbiota after undergoing pathological conditions (e.g., after antibiotic therapy). This is associated with an increase in bifidobacteria and a decrease in enterobacteria [185], which in turn may also affect an individual’s behavior [186]. With a view to preventing the negative effects of omega-3 fatty acid deficiencies, it is recommended that they be taken regularly as part of the diet (e.g., by consuming oily fish 1–2 times a week) or by appropriate supplementation, which should provide at least 250 mg of omega-3 fatty acids per day. It turns out that people who regularly consume fish are less prone to depression, which is explained by an increase in the output of serotonin that the body has already produced [187]. Similarly, supplementation with omega-3 fatty acids in the form of fish oil as part of the treatment of depression in adults has already contributed to a reduction in its symptoms after 8 weeks of use [188]. Moreover, seniors who regularly consumed about 300 g of fish per week (>3 times per week), compared to those who never or very rarely ate fish dishes, had about a 66% lower risk of depression, as measured by the Geriatric Depression Scale (GDS) [189]. Interestingly, the opposite effect was observed in those who ate breaded or deep-fried fish—in this group, the risk of increased depressive symptoms was higher [190].

Polyphenols are a diverse group of naturally occurring compounds found in plants, and they are known for their antioxidant properties and potential health benefits, including neuroprotection. In a study by Taram et al. (2016), the neuroprotective effects of chlorogenic acid (CGA) and its main metabolites—caffeic acid, ferulic acid, and quinic acid—were investigated in a rat neuronal primary cell culture exposed to various neurotoxic stressors. The study demonstrated that CGA and caffeic acid significantly protect neurons from the nitrosative stress induced by sodium nitroprusside. Furthermore, caffeic acid and ferulic acid were found to provide significant protection against the excitotoxicity invoked by glutamate. Notably, caffeic acid demonstrated protection across a wide spectrum of stressors, including oxidative stress induced by hydrogen peroxide, excitotoxicity, intrinsic apoptosis, endoplasmic reticulum (ER) stress, and proteasome inhibition [191].

Baicalin, a flavonoid compound extracted mainly from the root of *Scutellaria baicalensis*, a Chinese medicinal herb, has been shown to manifest neuroprotective and cognitive-enhancing effects. These include antioxidant stress, anti-excitotoxic, anti-apoptotic, and anti-inflammatory impacts, as well as promoting neurogenesis and the expression of neuronal protective factors. Baicalin has shown promise as a compound for supporting treatment in ischemic stroke, Alzheimer’s disease, and Parkinson’s disease, and has also demonstrating anti-anxiety and antidepressant effects [192].

Curcumin, the main active ingredient in the spice turmeric, is another subject of research regarding its impact on the mechanisms underlying anxiety and depression. Studies have demonstrated the efficacy of curcumin in influencing neurotransmitter levels, inflammatory pathways, excitotoxicity, neuroplasticity, HPA axis disorders, insulin resistance, oxidative and nitrosative stress, and the endocannabinoid system, all of which are potentially involved in the pathogenesis of MDD [193].

A recent study examined the liquiritin apioside in the context of inflammatory bowel disease (IBD), a condition known to contribute to depression in patients. Liquiritin apioside, the major flavonoid compound of licorice, was investigated for its mechanism of action in IBD, specifically focusing on its regulatory effects on metabolites derived from the gut microbiota and Th17 and Treg cell balance in mice with colitis. The results showed that liquiritin apioside treatment significantly improved the overall condition of mice with colitis and alleviated the associated depressive behavior. Liquiritin apioside was found to reduce the expression of pro-inflammatory cytokines, regulate Treg/Th17 differentiation, and increase the production of SCFAs in inflamed colonic tissues. Importantly, transplantation of fecal microbiota from liquiritin apioside-treated mice also improved the Treg/Th17 balance, highlighting the role of intestinal metabolites in the mechanism of action of this compound [194].

p-Hydroxycinnamic acids (HCAs), including ferulic, caffeic, sinapic, and p-coumaric acids, which are linked with various plant cell wall constituents, such as mono-, di-, and polysaccharides, sterols, polyamines, glycoproteins, and lignins, are significant dietary phenolic compounds. Intestinal microbes play a crucial role in releasing HCAs from these components, allowing for their absorption in free forms in the gut. Upon ingestion, HCAs are absorbed and undergo specific metabolic reactions within the small intestine or liver. HCAs contribute to the maintenance of epithelial barrier integrity and overall gut health through four main mechanisms: protection of TJ proteins, modulation of pro-inflammatory cytokines, exertion of antioxidant activities, and regulation of the gut microbiota. This multifaceted role underscores the significance of HCAs in the diet for supporting intestinal health and preventing dysbiosis [195].

## 4. Conclusions

Microbiota is an important link related to digestion and nutrient absorption, but it also exhibits a significant impact on the development of psychiatric disorders, especially those related to stress-relaxation and increased anxiety [196].

Despite numerous studies on GBA communication, the exact mechanisms of action of this pathway is still not fully understood. Many analyses show that there is a significant influence of the gut microbiota on the regulation of brain function and the nervous system [197]. The mechanisms involved in analyzing the importance of microorganisms and their metabolites, which can have a significant impact on brain physiology and nervous system function, are currently being studied. An important aspect in this field of research is the HPA, which is part of the body’s response to both physiological and physical stress [198].

Changes in the human diet, including dietary patterns, habits, and food processing, have greatly influenced gut health. Moreover, modern life and its impact on the gut microbiome have also made fundamental changes to the spectrum of human illnesses, shifting focus from traditional infectious diseases towards increasingly frequent mental diseases, such as depression. Ensuring a healthy diet and maintaining optimal intestine function are paramount for mental well-being, given the strong correlation between gut health and mental state. In the context of psychiatric disorders, especially among treatment-resistant patients, there is a growing interest in a holistic approach that acknowledges the impact of gut microbiota on mental health. Integrating probiotics into daily consumption emerges as a crucial aspect of this modern strategy, as they can support gut microbial balance and play a role in the holistic treatment of mental conditions, particularly in cases where conventional therapies prove ineffective. Thus, probiotics might serve as an additive therapy for depression.

## Figures and Tables

**Table 1:** Overview of selected bacteria and their impact on gut and brain function.

Bacteria	Effects on Intestines	Effects on the CNS
*Bifidobacterium breve*	Regulation of the gut microbiota composition [123].	Positive impacts on cognitive function, neuroprotective effect, and improvement of synaptic plasticity [123,124].
Lactic acid bacteria (LAB)	Inhibition of gut pathogenic bacteria, maintenance of gut barrier integrity, and homeostasis [125]. Regeneration of epithelial cells [126].	Reduction in neuroinflammation [127].
*Lactobacillaceae* and *Bifidobacterium*	Enhancement of the integrity of the intestinal epithelial barrier by increasing the levels of junction proteins and beneficially influencing the gut microbiota [128].	Improvement in memory and regulation of emotional behaviors by increasing the level of GABA in the hippocampus and regulation of central GABA receptor expression [129,130].
*Lactobacillus*	*Lactobacillus plantarum* 299v and *Lactobacillus rhamnosus* GG suppress inflammation and protect against intestinal barrier damage through regulatory effects on LPS-mediated cytotoxic activity in intestinal epithelial cells [131].	*Lactobacillus plantarum* 299v supports selective serotonin reuptake inhibitors (SSRIs) treatment, resulting in improved cognitive function and decreased kynurenine levels [132].
*Escherichia coli* Nissle 1917	Regulation of gut microbiota composition and amelioration of colonic barrier function [133,134].	Reduction in the severity of experimental autoimmune encephalomyelitis (EAE) connected with modulation of the inflammatory response of CD4+ T cells, their migration to the CNS, and restoration of the intestinal barrier integrity [135]. Neuroprotection, improvement in motor deficits, and reduction in brain inflammation [134]. Reductive impact on anxiety-like behaviors [133].
*Akkermansia muciniphila*	Acceleration of intestinal stem cell proliferation and cell differentiation in the gut [136]. Activation of NF-κB in intestinal cells, enhancement of barrier function and immune responses [137]. Reduction in the severity of acute colitis symptoms and inflammation [138].	Inhibition of inflammatory cytokines in microglial cells [139]. Reduction in depressive-like behaviors and modulation of gut serotonin dynamics in mice [75].
*Roseburia hominis*	Immunomodulation [140].	Reduction in neuroinflammation via histone deacetylase inhibition [141].
*Clostridium butyricum*	Protection of the gut barrier integrity, a decrease in the levels of D-lactate in plasma and IL-6 in the colon, and up-regulation of occludin expression [142].	Neuroprotective effects against neurological dysfunction, brain edema, neurodegeneration, and BBB disruption [142].
*Faecalibacterium prausnitzii*	Enhancement in intestinal cell health and reduction in inflammation [143]. Improvement in gut barrier integrity [144,145].	Improvement in cognitive impairment in a mouse model of Alzheimer’s disease [146].

**Table 2:** Overview of clinical evidence supporting the psychobiotic properties of some bacterial strains.

Form, Bacterial Strain	Dose	Effects
Postbiotic, *Bacillus coagulans* MTCC 5856	2 trillion spores	Effective in treating patients with IBS symptoms who had been diagnosed with depression [165].
Probiotic, *Bifidobacterium longum* 1714	1 × 10^9^ CFU/d	Reduced stress symptoms and improved memory [166].
Probiotic, *Bifidobacterium longum* NCC3001	1 × 10^10^ CFU/g	Reduced symptoms of depression and reduced reactions to negative emotional stimuli [167].
Probiotic, *Clostridium butyricum* MIYAIRI 588	60 mg/d	When combined with antidepressants, this strain was effective in treating drug-resistant depression [168].
Probiotic, *Lactobacillus casei* Shirota	1 × 10^9^ for 8 weeks	Reduced perceived stress [169].
Probiotic, *Lactobacillus gasseri* CP2305	1 × 10^10^ CFU	Reduced reactions to stressful situations and improved sleep quality [170].
Multi-strain probiotic supplement, *Bifidobacterium bifidum* W23, *Bifidobacterium lactis* W52, *Lactobacillus acidophilus* W37, *Lactobacillus brevis* W63, *Lactobacillus casei* W56, *Lactobacillus salivarius* W24, *Lactococcus lactis* (W19 and W58)	2.5 × 10^9^ CFU/g	Reduced susceptibility to lowered mood states [171].
Multi-strain probiotic supplement, *Lactobacillus acidophilus*, *Lactobacillus casei*, *Bifidobacterium bifidum*, *Lactobacillus fermentum*	1 capsule contained 2 × 10^9^ CFU/g per day, administered for 12 weeks	Improved overall health; reduced anxiety symptoms and depressive conditions. Reduction in inflammation in the body [172].
A fermented beverage made from black soybeans, *Lactobacillus gasseri* CP2305	190 g serving once a day for 5 weeks	Improved sleep quality and reduced stress-related symptoms in healthy adults [170].
A fermented beverage made from black soybeans, *Lactobacillus casei* Shirota	100 mL serving once a day for 8 weeks	Increased serotonin levels in the body. Reduced stress symptoms in people exposed to stressful situations [173].
Probiotic milk drink, *Lactobacillus acidophilus*, *Lactobacillus casei*, *Bifidobacterium bifidum*, *Lactobacillus fermentum*	200 mL serving once a day for 12 weeks	Positive effects on cognitive function in patients with Alzheimer’s disease (aged 60–95 years) [174].
Fermented soybean seed paste, *Lactobacillus plantarum* C29	800 mg per day for 12 weeks	Improved cognitive function in people with mild cognitive impairment [175].
Unpasteurized milk and dairy products, *Lactobacilli*	Unlimited consumption for 12 weeks	Reduced stress reactions and anxiety in adults [176].
Probiotic yogurt, *Lactobacillus acidophilus* LA5 and *Bifidobacterium lactis* BB12	100 g serving once a day for 6 weeks	Positive impact on depression treatment; improvement in symptoms of depression, anxiety, and stress among adults [177].
Yogurt, Lactobacillus gasseri SBT2055 and Bifidobacterium longum SBT2928	100 g once a day for 12 weeks	Decreased levels of stress-induced hormone adrenocorticotrophic hormone [178].

## Data Availability

The data used in this article are sourced from materials mentioned in the References section.
